# Stress: Eight Decades after Its Definition by Hans Selye: “Stress Is the Spice of Life”

**DOI:** 10.3390/brainsci13020310

**Published:** 2023-02-12

**Authors:** Luc Rochette, Geoffrey Dogon, Catherine Vergely

**Affiliations:** “Pathophysiology and Epidemiology of Cerebro-Cardiovascular Disease” Research Unit (PEC2, EA 7460), University of Burgundy and Franche-Comté, UFR des Sciences de Santé, 7 boulevard Jeanne d’ Arc, 21000 Dijon, France

## 1. Introduction

July 1936: Hans Selye describes in 74 lines in the prestigious journal *Nature* a new concept: Stress [1]. Eighty years later, stress is an integral part of our lives. Hans Selye who wrote “Stress is the spice of life” is the founding father of the concept of general adaptation syndrome. His notoriety earned him the title of “Einstein of Medicine”. In the journal *Nature*, which he titled: “A Syndrome Produced by Diverse Nocuous Agents”—Selye had written “noxious” but the editor replaced this word with “nocuous”; similar levels of toxicity but of different intensities.

Born in Vienna in 1907, where his father, of Hungarian origin, was a doctor, Hans Selye studied in Komarom, the town where his parents lived after the end of the 1914–1918 war. His family and educational environment predisposed him to speak several languages. He studied at the German University in Prague where he obtained a doctorate in medicine (1929) and a doctorate in organic chemistry (1931). He later chose research over practice, working at the Institute of Pathology in Prague. He made stays in Paris where he was impressed by the laboratory of Marie Curie and in Rome. He became a Rockefeller Foundation Scholar in 1931, which led him to study in the United States at the Johns Hopkins University (1931) and then in Canada at the McGill University (1931–1934).

The McGill University appointed him an Assistant Professor of biochemistry (1934–1941) then Associate Professor of histology. He obtained Canadian citizenship in 1939. In 1945, he went to the University of Montreal where he became a Professor in the Faculty of Medicine and founded the Institute of Experimental Medicine and Surgery, of which he became Director. He held this position until his retirement in 1977. He founded the International Institute for Stress in Montreal. A Companion of the Order of Canada, he died in Montreal in 1982. Hans Selye published more than 1700 articles and 39 books on stress.

In several of his works, including “*The Stress of Life*” [2], Selye reports the difficulties and hesitations for the choice of the word: stress. He evokes in particular his conference at the Collège de France in Paris during 1946 when he was invited to speak about the general syndrome of adaptation. What emotion for him to be the speaker, where many years before, Claude Bernard developed the concept of the “inner environment”. He then evoked his hesitation to use the English word: stress. The French words: aggression, tension or distress partially reflect the concept of stress. In front of the professors of the Collège de France he adopted the masculine gender and yet aggression, tension and distress are feminine words in the French language. He noted that some French authors then used approaches such as stimulation or aggression and that Germans translated it as schaden (damage) and Spanish as sufrimiento (suffering).

In 1956, his work which would soon make him famous, “*The Stress of Life*” was published. The Editor of the book wrote: “this book is written by one of the outstanding men of our time—a man who may well rank with Pasteur and Ehrlich in medical history”. His theories on the body’s response to physical, environmental or psychological agents revolutionized our understanding of the causes and mechanisms of disease, as well as the links between the brain, emotions and the body. He elaborated on the concepts of coping energy, stress management, post-traumatic coping, and behavioral codes that protect against exposure to “harmful” environments. “The idea for the concept of stress and the general adaptation syndrome came to me in 1925, when I was studying medicine at the University of Prague. […] I had stuffed myself with the maximum of theoretical knowledge and I was burning with enthusiasm for the art of healing […]. What struck me, as a novice, was that apparently very few symptoms characterized each disease; […] I could not understand why, from the dawn of the history of medicine, doctors concentrated all their efforts on the recognition of particular diseases and the discovery of specific remedies without giving any attention to something more obvious: “the syndrome of the simple fact of being sick”. Would it be possible to analyze this “general syndrome of the disease” and perhaps even find remedies capable of acting against the non-specific factor(s) of the disease?

Selye was very clear about the stages that characterize the stress syndrome or general adaptation syndrome. It evolves in three successive stages: initially: the “alarm reaction” [3] during which the defense forces are mobilized; it is the stage of appearance of acute manifestations which cease only with the disappearance of aggression; then the “stage of resistance” sets in which reflects complete adaptation to the stressor; and finally the “stage of exhaustion”; characterized by the depression of organic functions.

This characterization of the chronology of the stages of stress is based on experimental results. In 1935 in Montreal, he was working as an Assistant in the biochemistry department, on sex hormones. He had to inject rats with different ovarian and placental extracts, looking at the short-, medium-, and long-term effects. He noted the famous triad: (1) hypertrophy of the adrenal cortex; (2) atrophy of the thymus, spleen, and lymph nodes; (3) stomach and intestinal bleeding and ulcers. Selye observed that this triad of modifications formed a well-defined syndrome, since these alterations were closely linked and their intensities were proportional to the quantity of tissue extracts injected.

Selye discussed in these comments the importance of the duration of the different stages: alarm stage: 6–48 h, the second stage begins after 48 h. He reported the involvement of the brain–periphery axis: the axis between the cerebral spheres, the pituitary, and the endocrine glands [3]. It describes the potential role of information “mediators” between brain territories and organs, as well as the conditions for the activation of hormones. Stress being the non-specific response given by the body to any request made to it, it became essential to clarify the conditions of stress initiation; it was then necessary to define the “stressors”. Whether the stressor is pleasant (pleasure) or not (desperation) is irrelevant, its effect depends on the “intensity of demand” made on the body’s “adaptive capacity”. These “stressors” need to be integrated into the overall scheme of the adaptation/maladaptation balance and adaptation disorders [4].

In the chronology of the spatial and temporal concept of adaptation, Selye’s scientific approach is very far-sighted [5]. He then described the essential role of the inflammatory response in any process of tissue damage. The biological processes that participate in stress will oppose the permanence of the inflammation in different ways, in particular by soliciting the adrenal cortex—endocrine glands that secrete corticosteroid hormones with anti-inflammatory properties.

Selye, throughout his career, identified the different components that participate in the installation and development of stress. As part of the links between general adaptation syndrome and local adaptation syndrome, he then referred to a new concept, that of “adaptive energy”. Human life expectancy is determined by the availability of energy during adaptive processes. In the development of his thought, he oriented his reflection towards “the evolution of an intercellular altruism” during embryogenesis with “communities of interest” [6].

Hans Selye, the “Einstein of Medicine”, did not see his work crowned with a Nobel Prize, although nominated ten times, which regrets one of his students Nobel Prize in Medicine and Physiology (1977): Professor Roger Guillemin from Dijon in France and living in San Diego: “To Professor Hans Selye, to whom I owe my training in experimental medicine, I present this work which would have been impossible without his affectionate confidence and the proximity of his astonishing scientific genius”, this is the dedication when opening the thesis of Roger Guillemin defended in Montreal in 1952 [7].

Eight decades after the initial description of the concept of stress, stress has diversified and now concerns specific areas of biology, such as stress involving oxygen oxidative stress [8,9] and psychological stress [10]. More than 130,000 articles are available to us to read, enough to amplify or reduce our stress!

## Data Availability

Not applicable.
